# A Possible Role of Dust in Resolving the Holocene Temperature Conundrum

**DOI:** 10.1038/s41598-018-22841-5

**Published:** 2018-03-13

**Authors:** Yonggang Liu, Ming Zhang, Zhengyu Liu, Yan Xia, Yi Huang, Yiran Peng, Jiang Zhu

**Affiliations:** 1 Department of Atmospheric and Oceanic Sciences, School of Physics, Peking University, Beijing, 100871 China; 2 Atmospheric Science Program, Department of Geography, Ohio State University, Columbus, Ohio, 43210 USA; 3 Department of Atmospheric and Oceanic Sciences, McGill University, Montreal, Quebec, H3A 0B9 Canada; 4 Department of Earth System Science, Tsinghua University, Beijing, 100084 China; 5 Department of Earth and Environmental Sciences, University of Michigan, Ann Arbor, MI 48109 USA

## Abstract

Climate models generally fail to produce a warmer (by as much as 0.5 °C) early to mid-Holocene than the pre-industrial in the global annual temperature, which has been termed the Holocene temperature conundrum. Here we use a fully coupled atmosphere-ocean general circulation model to test whether the conundrum can be partially resolved by considering the fact that atmospheric dust loading was much reduced during the early to mid-Holocene. Our experiments show that the global annual mean surface temperature increases by 0.30 °C and 0.23 °C for the mid-Holocene (6 ka) and early Holocene (9 ka), respectively, if the dust is completely removed. The temperature increase scales almost linearly with the fraction of dust being removed, with the 50% dust reduction experiment for the 6 ka being the only one deviating from the linear trend. The indirect effect of dust, which is highly uncertain and is not included in the model, may further enhance the warming. Therefore, the neglect of dust reduction in the Holocene in climate models could contribute significantly to the model-data discrepancy, especially in the Northern Hemisphere.

## Introduction

A recent reconstruction of global annual mean surface temperature in the Holocene shows a cooling trend from the peak warming of ~0.5 °C in the Holocene Thermal Maximum (HTM) (~10–6 thousand years ago; ~10–6 ka) towards the pre-industrial (PI)^1^. This cooling trend is opposite to current transient climate simulations, which show a robust warming trend forced mainly by the retreat of the residual ice sheet and the increase of greenhouse gases (GHG)^2^. Moreover, snapshot experiments of the 6 ka climate by 31 atmosphere-ocean general circulation models (AOGCMs) participated in the Paleoclimate Modeling Intercomparison Project phase II (PMIP2) and phase III (PMIP3) mostly produced a cooler or negligibly warmer climate than the PI (Supplementary Fig. 1). The cause of this apparent model-data inconsistency, termed the Holocene temperature conundrum^2^, has remained elusive. Here, we propose that the neglect of mineral dust response in climate models could contribute significantly to this discrepancy. Using a climate model, we show that the reduced atmospheric dust loading, which can be generated mainly by an enhanced monsoon and in turn vegetation cover in the North Africa during the mid-Holocene, could generate a global annual mean warming up to 0.3 °C. This model bias, when combined with the potential seasonal bias of the proxy record, can reduce the model-data discrepancy significantly.

**Figure 1:**
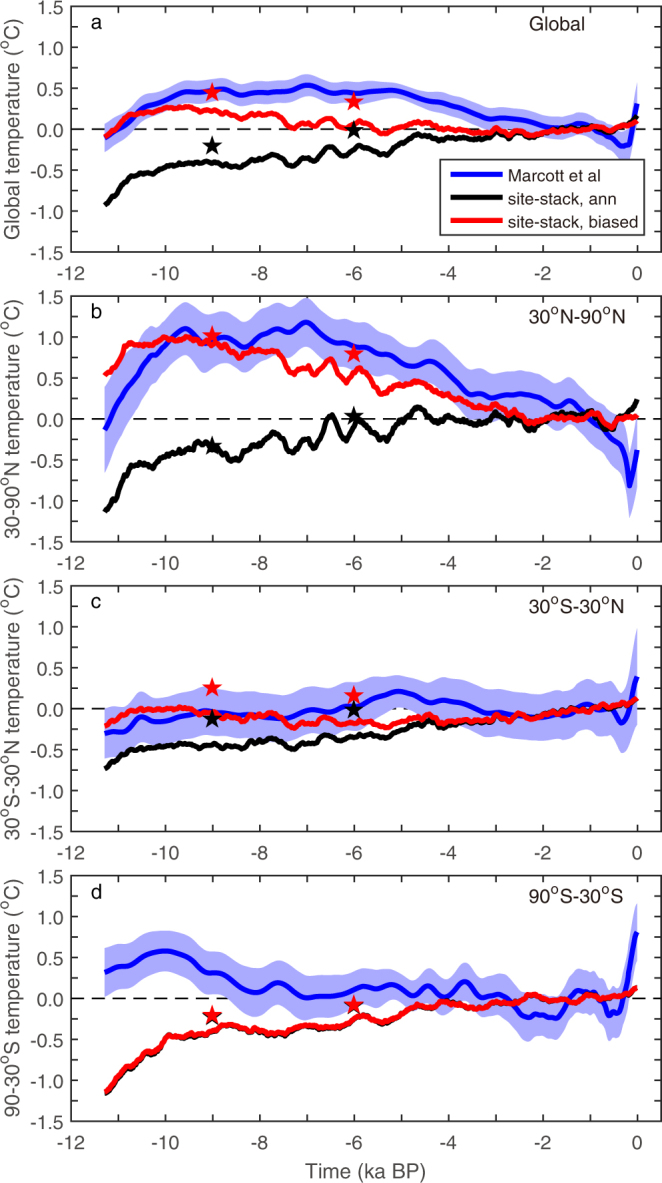
Modified from Fig. 3 of Liu *et al*.^2^. The standard 5° × 5° weighted temperature stack for the last 11 kilo-years from the proxy dataset (blue curves)^1^ and similarly stacked global and regional mean temperatures from transient simulations (black and red curves). The simulation results shown here are the ensemble mean of three models: CCSM3, LOVECLIM and FAMOUS, with both the site-stacked annual mean (black) and site-stacked seasonally biased (red). (**a**–**d**) are the global mean, Northern Hemisphere (30–90° N) mean, tropical (30° S–30° N) mean, and Southern Hemisphere (90–30° S) mean, respectively. The reference climate is the average between 1.5 ka and 0.5 ka. The change of surface temperature due to dust removal at 6 ka and 9 ka are indicated by pentacles.

It has been suggested that one potential cause of the model-data discrepancy is the summer bias in some temperature proxies^3^. This bias can contribute to a warming in HTM period due to the increased summer insolation in the Northern Hemisphere^2^. Taking into account of the summer bias in proxy records, transient climate modeling can indeed generate a global cooling trend as in the reconstruction, especially in the Northern Hemisphere (red lines in Fig. 1a,b). However, summer bias in the proxy data cannot account for all the discrepancies. First, even with this summer bias, models still underestimate the warming during the HTM period, both in the transient simulations (Fig. 1) and in the snapshot simulations of PMIP models (Supplementary Fig. 1b). Second, summer bias cannot explain the observed warming in the Southern Hemisphere (Fig. 1d). Indeed, an austral summer bias would lead to a cooling in the HTM period in the SH because of the reduced summer insolation there, leading to an even greater model-data discrepancy. It should also be noted that, since most of the data here^1^ are reconstructions of the sea surface temeprtures, the model-data discrepancy over the proxy sites can’t be reconciled as in the land-based proxy reconstruction from North America and Europe^4^. Therefore, additional mechanisms are needed to reconcile the model-data discrepancy. One potential mechanism is the model bias associated with the changing mineral dust, which has been neglected in previous transient model simulations that were used in the study of the Holocene conundrum^2^.

In the HTM period, the stronger boreal summer insolation leads to a significant enhancement of monsoon and, in turn, vegetation cover in the Northern Hemisphere^5,6^. Especially over North Africa, during the African Humid Period (AHP; 14.8–5.5 ka)^7–10^, shrub and grassland expanded greatly over the Saharan desert^11,12^, which should lead to a great reduction of dust emission and, in turn, dust loading in the atmosphere.

Marine sediment records along the northwest African coast in the Atlantic all show substantial decrease of dust output from Africa during AHP^13–16^. A few modeling studies have attempted to simulate the dust cycles during the mid-Holocene using general circulation models^17–20^. There is still large uncertainty in modeling the dust cycle^21^. For example, the global dust loading at 6 ka simulated by Albani *et al*.^17^ using the Community Earth System Model (CESM) was only ~25% weaker than that during the preindustrial, although it has been recognized that this dust emission from the North Africa seems to be overestimated compared to sedimentary records^17^. The dust deposition flux simulated by Egerer *et al*.^18^ seemed to fit the sedimentary cores well, their study suggested that the dust emission flux from North Africa during mid-Holocene was 27% of the preindustrial and the global dust loading was ~38% of the preindustrial.

Climate modeling indicates that the Atlantic ocean temperature is sensitive to local change of dust aerosol, and dust reduction may be able to explain 69% of the upward trend observed between 1982–2007^22^. By reducing the dust loading over tropical North Atlantic region by 50% (while keeping the loading elsewhere), Williams *et al*.^15^ showed that the same region would warm by a fraction of a degree under present-day climate condition. There have also been studies on the dust effect in the Holocene, mostly on the regional response in African monsoon and nearby region monsoon^23^ or ENSO^24^. There has, however, been no systematic study on the effect of dust reduction on global surface temperature in the Holocene.

Here we use a fully coupled atmosphere-ocean general circulation model, CCSM3, to test the influence of dust reduction on the early Holocene (9 ka; EH) and mid-Holocene (6 ka; MH) climate. The atmospheric dust loading is reduced by different amounts in a series of simulations. The model version and resolution are chosen to be the same as that used by Liu *et al*.^2^ in simulating the transient climate evolution from LGM to PI. Therefore, the response of global surface temperature to dust reduction obtained here may serve as a correction to that obtained in Liu *et al*.^2^, to see whether it helps to resolve the Holocene temperature conundrum.

## Results

### Mechanism of warming due to dust reduction

**Figure 2:**
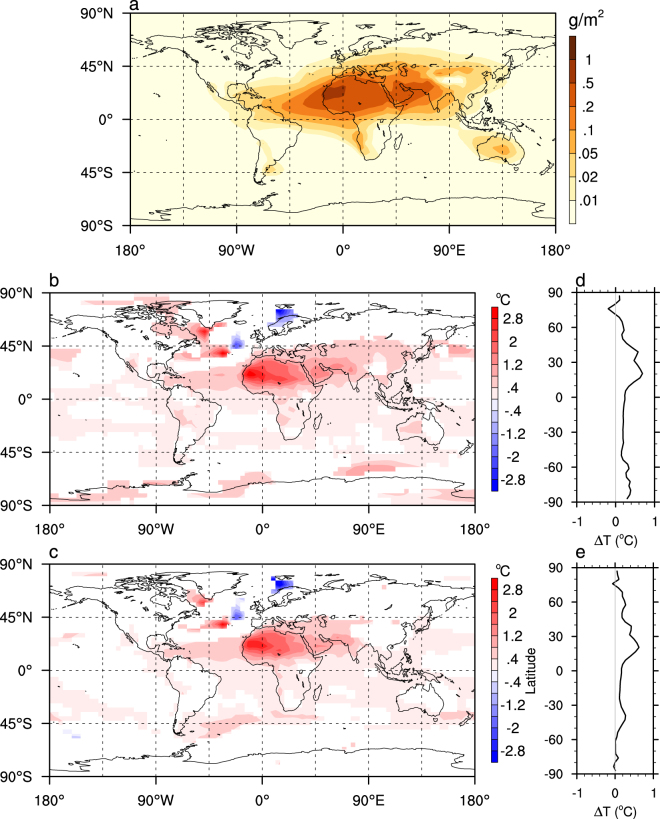
Annual-mean present-day dust loading (**a**), and the surface temperature change in (**b**) 6 ka and (**c**) 9 ka when this dust is completely removed. Only the changes that are significant to the 5% level (calculated by two-tailed student-t test) are shown. The zonal-mean temperature change in (**b**,**c**) are shown in (**d**,**e**), respectively. Figure generated using NCL^46^.

**Figure 3:**
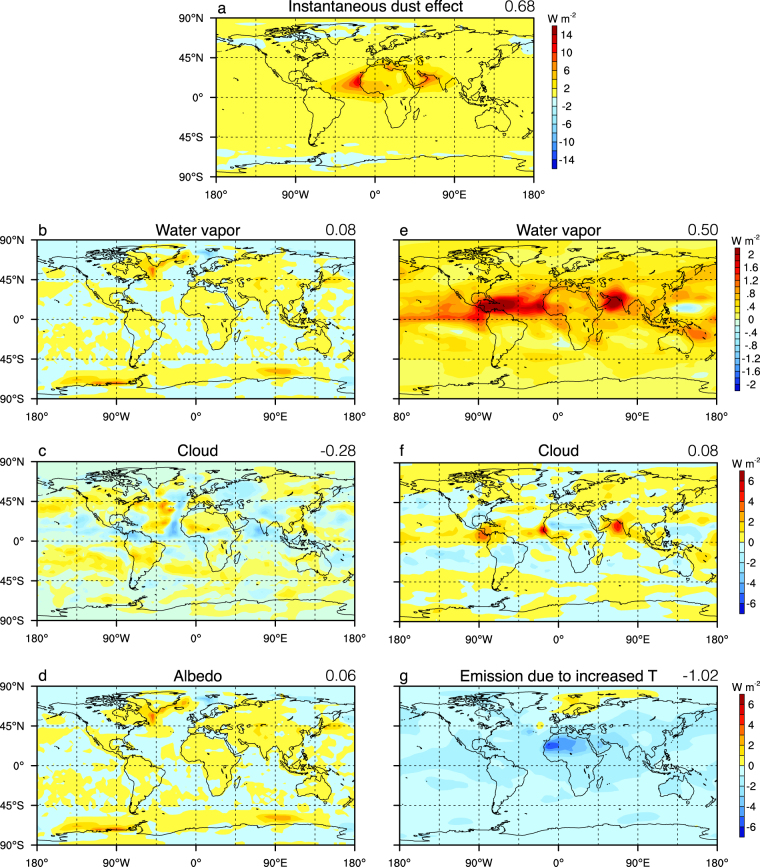
Change of shortwave (**a**–**d**) and longwave (**e**–**g**) top-of-atmosphere radiative fluxes due to different components for the MH climate when dust is completely removed. In g), the increased outward longwave emission is due to temperature increase of both surface and atmosphere. The global mean values are indicated at the top right corner of each panel, with unit of W m^−2^. Note the different color scale for each row. Figure generated using NCL^46^.

When dust is completely removed, annual mean surface temperature over northern Africa, Arabia and Iran is increased significantly, by as much as 2.5 °C, and that over the tropical North Atlantic is increased by ~0.5 °C for both the MH and EH climate (Fig. 2b,c). These regions (focusing on the MH climate first) are under direct effect of dust removal, and are warmed mainly by the increased shortwave forcing (Supplementary Fig. 3a). The longwave forcing at the top of atmosphere (TOA) over these regions is negative (i.e. more is going out) but of smaller magnitude (Supplementary Fig. 3b). The change of the net radiative fluxes at TOA is decomposed into the instantaneous radiative effect of the dust and radiative feedbacks consisting of the water vapor, cloud, albedo, and temperature feedbacks^25,26^ (Fig. 3). The TOA radiative kernels of Shell *et al*.^27^, created from the NCAR CAM3, are used here. The analyses show that the increased shortwave forcing is primarily due to reduced scattering of dust (Fig. 3a). Averaged globally, the direct radiative forcing of dust removal for the MH climate is 0.68 W m^−2^. This value is similar to total direct forcing (0.60 W m^−2^) for the present-day climate in a more advanced version of CCSM3 that calculates the longwave radiative effect of dust, diagnosed by Yoshioka *et al*.^28^ using a more accurate but computationally expensive method.

The positive feedback due to water vapor, 0.58 W m^−2^ (Fig. 3b,e), is of almost the same magnitude as the direct forcing of the dust. The response of cloud in this model generates a net negative forcing of −0.20 W m^−2^ (Fig. 3c,f). The change of longwave forcing corresponds well to the change of high cloud, while the shortwave forcing corresponds mostly to the change of middle cloud in the tropical region and to the change of low cloud in the extratropics (Supplementary Fig. 4). The heat transports in the atmosphere and ocean tend to compensate with each other in the mid- to high latitude^29^, but they both transport heat away from the northern tropical region where the most of the dust is removed (Supplementary Fig. 5). The equivalent radiative forcing of heat transport averaged over the southern hemisphere (90–30°S) is 0.23 W m^−2^, much larger than the direct forcing of dust (0.08 W m^−2^) there. This heat transport is mainly achieved by the atmosphere (Supplementary Fig. 5a). The heat transport enhances sea-ice melting in both polar regions, which induces a further positive feedback by reducing surface albedo (Fig. 3d). When averaged over the globe, this generates a small positive forcing of 0.06 W m^−2^. The positive forcings above are approximately balanced by the increased emission of longwave radiation due to increased surface and air temperature (Fig. 3g; see all the numbers in Fig. 3). As a result, the global mean surface temperature increases by 0.30 °C. The cooling in the Nordic seas (Fig. 2b) is caused by slight weakening of the Atlantic meridional ocean circulation (AMOC). If a slab ocean of 50 m thick instead of a dynamical ocean was used in the model, the surface temperature would increase almost everywhere when dust is removed (Supplementary Fig. 6). The change of global mean surface temperature is 0.31 °C, almost the same as that in the coupled model. Therefore, the major role of the ocean dynamics is to distribute heat differently, most notably in the northern North Atlantic (compare Supplementary Fig. 6 with Fig. 2b).

For the EH, removal of dust induced a global warming of 0.23 °C, less than that for the MH, mainly due to more negative cloud forcing and smaller water vapor feedback (Supplementary Fig. 7). Because the instantaneous direct forcing of dust is diagnosed to be the same between EH and MH (compare Supplementary Fig. 7a and Fig. 3a), this indicates dependence of dust effect on the background climate state rather than on the variation of seasonal insolation.

### Towards reconciling model-data discrepancy

Our model derived warming due to dust removal can be used to estimate the potential contribution of the dust effect on model-data discrepancy. In particular, since the model employed here is the same as that used in the CCSM3 transient simulation in Liu *et al*.^2^, our results can be considered as a direct correction to the CCSM3 transient simulation there with the dust effect. Our model estimation indicates that the model-data discrepancy may be significantly reduced had the reduction of dust emission during AHP been considered in their simulations. For the extreme case of a complete removal of dust, the increase of MH annual temperature (seasonally-biased site stack) is 0.10 °C (0.26 °C), 0.33 °C (0.32 °C) and 0.15 °C (0.15 °C) averaged over the northern hemisphere (30–90°N), tropics (30°S–30°N) and southern hemisphere (90–30°S), respectively. This magnitude of warming almost compensates the model discrepancy at 6ka, if the summer bias on proxy data is considered, for the northern hemisphere and tropics, and in turn global mean (red stars, Fig. 1). The improvement in the southern hemisphere, where the model-observation discrepancy could not be explained by seasonal bias of the proxy data at all, is also obvious, although the data-model discrepancy remains large (Fig. 1d).

For the EH, the model-data discrepancy is similarly reduced in terms of global mean when dust is removed (Fig. 1 and Supplementary Table 1). Regionally, the temperature over the tropical region is over-corrected (Fig. 1c), while the southern hemisphere under-corrected (Fig. 1d). However, the snapshot simulation for the early Holocene done here may be less appropriate than for the mid Holocene because the climate has significant transient evolution during the early Holocene (black lines in Fig. 1). The response of southern ocean to dust reduction in a transient simulation may differ from that simulated here; it may transport more heat to the polar region when dust is removed, rather than less heat as simulated here (Supplementary Fig. 5b). Of course the large model-data discrepancy in the southern ocean (Fig. 1d) could as well be due to insufficient knowledge about the evolution of the Antarctic ice sheet during the last deglaciation; the freshwater forcing in the southern hemisphere applied in the CCSM3 simulation of Liu *et al*.^2^ (see Fig. 25a of He^30^) is much different from the time series of iceberg discharge from Weddell Sea reconstructed more recently^31^.

**Figure 4:**
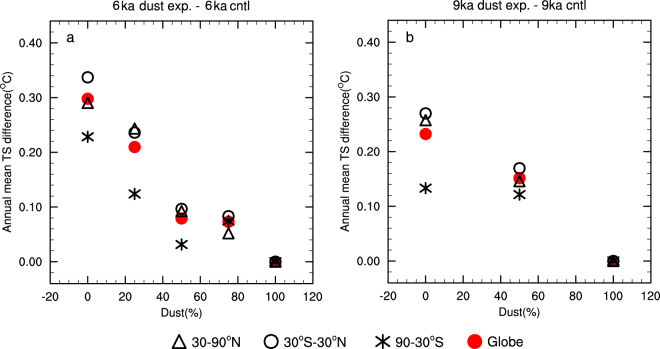
Change of global-mean annual surface temperature for different dust loading relative to that for the default (100%) dust loading, for (**a**) 6 ka and (**b**) 9 ka. Different symbols represent mean values over the northern hemisphere (triangle; 30–90°N), tropics (circle; 30°S–30°N), southern hemisphere (asterisk; 90–30°S), and the whole globe (red filled circle).

The change of global-mean annual surface temperature is smaller when only a fraction of dust loading is removed. It increases linearly by 0.030 °C (0.023 °C) per 10 percent of dust removal with a correlation coefficient of 0.97 (0.99) for the MH (EH) (Fig. 4). The site-stacked global-mean temperature also scales linearly because the patterns of temperature change are similar for different dust reductions (not shown). Therefore, if the dust loading in the atmosphere was reduced by ~60% as estimated in ref.^18^ (see the Introduction section) instead of 100%, during the AHP, the global-mean surface temperature increases by 0.18 °C (0.17 °C for the seasonally-biased site-stack). The warming effect for the 50% dust reduction in the MH appears anomalous because it is the only experiment that deviates from the overall linear relationship in both MH and EH (Fig. 4). Because of this, an additional CCSM3 simulation was carried out to determine the warming effect of a 60% dust reduction more precisely, which gave a result of 0.14 °C. The ensemble mean of PMIP2 and PMIP3 snapshot experiments produced an MH climate that is 0.16 °C (seasonally-biased site-stack) warmer than the PI (the red pentacle in Supplementary Fig. 1). If we take the model-data discrepancy to be 0.50–0.16 = 0.34 °C, a 60% reduction of dust loading can therefore still account for about half of the remaining model-data discrepancy.

## Discussion

Our results suggest that the Holocene conundrum can be contributed significantly by biases in both data and model. Some temperature proxies have summer biases, while the models may have neglected the reduced mineral dust in the early to mid-Holocene. Our study remains preliminary. One large uncertainty in our study is the neglect of the indirect radiative effect of mineral dust aerosol. Part of the reason we didn’t include this is that the indirect effect has a very large uncertainty in current estimations^32^. Nevertheless, if we use an additional warming effect of 0.30 W m^−2^ (the difference in Net Effective Radiative Forcing between the two LD cases in Table 2 of Sagoo and Storelvmo^33^) due to indirect dust radiative forcing for a 90% reduction of dust loading (Table S1 of Sagoo and Storelvmo^33^), and assume it scales linearly with different fractions of dust reduction, the MH climate could warm by an additional 0.09 °C with a 60% dust reduction. This number is obtained based on the assumption that a 0.68 W m^−2^ forcing causes a global warming of 0.3 °C as described above. Therefore, the total warming would be 0.27 °C (or 0.23 °C if the more precise value for the 60% dust reduction is used, see the paragraph above) even with a 60% reduction of dust. Note that the estimated dust indirect radiative effect in Sagoo and Storelvmo^30^ extends in a certain range, which is attributed to the subtleties in ice cloud formation scheme and cloud feedbacks on climate in the global model (e.g., Table S2 of Sagoo and Storelvmo^30^). The effect is too complicated to discuss here, but is of significance for future studies.

Here, for clarity, we only focused on the dust effect alone; the change of vegetation and lake levels are not considered. In experiments in which the North Africa is changed to grassland, the change of global-mean surface temperature of MH (EH) due to complete removal of dust is 0.26 °C (0.23 °C), almost the same as in the dust-only experiments described above, with also similar response pattern (see Supplementary Table 1 and Supplementary Fig. 8). Therefore, the vegetation effect itself appears to be not significant relative to the associated dust effect in this model.

Finally, models have biases and uncertainties, and uncertainty in the radiative effect of dust is not small^32,34^. Especially, a recent study argued that the direct radiative forcing of dust should be lower than previously expected^35^. But it is possible that their results were biased due to their assumptions of spherical shape, constant mixing state and optical properties of dust^36^. Therefore, much work is awaited to determine the radiative effect of dust. A multi-model experiments will also be needed in the future to assess the robustness of the dust effect on the HTM climate. Overall, our study highlights the dust effect as a potentially important factor for understanding climate changes in the past and future. The identification of the model-data discrepancy is improving our understanding of both the model and data.

## Methods

**T**he fully coupled atmosphere-ocean general circulation model (AOGCM), Community Climate System Model version 3 (CCSM3)^37^ is employed here. Both the atmospheric module (CAM3) and the land module (CLM3) were run at a horizontal resolution of T31 (3.75° × 3.75°), and both the ocean (POP1.4) and sea ice (CSIM5) modules at “gx3v5” (with 100 and 116 grid points in the zonal and meridional directions, respectively). In the vertical, the atmosphere and ocean were divided into 26 and 25 levels, respectively. In this version of CCSM3, dust loading in the atmosphere is prescribed. The model calculates only the shortwave radiative effect of the dust, omitting the longwave effect and the indirect effect^28,34,38^. Most estimates show that the global-mean longwave effect is much smaller than the shortwave effect both at the surface^32^ and at the top of atmosphere^34^. The indirect effect of dust through its interaction with cloud (mainly on ice cloud^33,39–41^) may be large for the present day climate locally, but there is few estimate of its global influence.

The MH control climate was obtained by running the model with MH orbital parameters (at 6 ka), set according to the PMIP2 MH benchmark experiment^42^, while everything else, including Greenhouse gas concentrations and ozone, were prescribed the same as for PI. The only change for the EH control climate simulation relative to the MH climate simulation is the orbital parameters (from 6 ka to 9 ka). The climate effect of dust removal during the EH is assumed to be the same whether the remnant ice sheet is considered or not. This may not be a bad assumption because the orbital effect is larger than the effect of remnant ice sheet in CCSM3^2^. The present-day dust distribution is prescribed, which was produced using an aerosol assimilation system^43^ and released with CCSM3. The dust loading is mostly over northern Africa, Arabia, Iranian plateau and western India (Fig. 2a), and has the largest amplitude during boreal spring and summer (Supplementary Fig. 2). To test the effect of dust reduction on global surface temperature, the atmospheric dust loading is reduced uniformly by a factor of, 25%, 50%, 75% and 100% for the MH, and 50% and 100% for the EH. This uniform dust reduction is somewhat crude but is based on the following considerations: (1) there are both observational^44^ and modeling^42,45^ evidence that climate was wetter not only in northern Africa during MH, but also over Arabia and western India; (2) the dust deposition rate in the tropical and northern Atlantic, the Arabia Sea and east Asia all showed some depression over the mid Holocene relative to the late Holocene^17^; (3) the coupled dust and climate modeling could not provide consistent dust loading for the Holocene^17–19^; (4) the dust loading is much smaller over regions like Gobi desert, Australia and South America at least in the present day (Fig. 2a), decrease of which may not be climatically consequential.

The control and dust-reduction experiments for EH and MH are all initialized from the PI equilibrium climate state, and are run for 1000 years. Running the control and dust-reduction experiments in parallel allows early diagnosis of the dust effect and saves waiting time. It is found that the dust effect diagnosed around model year 400 and year 1000 has negligible difference. Sensitivity experiments show almost the same the results if the control experiment was run to equilibrium first and then initialize the dust-reduction experiment from this equilibrium state. Therefore, it is not necessary to run the experiments to equilibrium to diagnose the effect of dust, and experiments for non-100% reduction are run for 500 years. At that time, the surface temperature are drifting at rate of ~0.4 °C/1000 years for both the control and dust-reduction experiments. The last 50 years of data are used for analyses. The MH control experiment shows a global cooling of 0.5 °C from the PI of the same dust, within the range of the state of the art models in PMIP experiments (Supplementary Fig. 1).

### Data availability

The datasets generated during and/or analysed during the current study are available from the corresponding author on reasonable request.

## Electronic supplementary material

Supplementary information
