# Supplementary material for: A Possible Role of Dust in Resolving the Holocene Temperature Conundrum
Source: Sci Rep. 2018 Mar 13;8:4434. doi: 10.1038/s41598-018-22841-5 (PMC5849762; doi:10.1038/s41598-018-22841-5)
Supplement: Supplementary file 1 — Supplementary information [file 41598_2018_22841_MOESM1_ESM.pdf]

Supplementary Information for "**A Possible Role of Dust in Resolving the Holocene Temperature Conundrum**"

Yonggang Liu, Ming Zhang, Zhengyu Liu, Yan Xia, Yi Huang, Yiran Peng, and Jiang Zhu

Supplementary Table 1 The change of surface temperature due to complete dust removal (green North Africa and dust removal). The areal average of the temperature is calculated using three different methods: directly average over the model grid weighted by the grid cell area; construct a virtual site-stack using the model annual-mean surface temperature as done in *Liu et al.*, 2014[1]; construct a virtual site-stack using the seasonally biased model surface temperature as done in *Liu et al.*, 2014[1];

	MH (6 ka)				EH (9 ka)			
	Global	N. Hemi.	Tropics	S. Hemi	Global	N. Hemi.	Tropics	S. Hemi.
Model grid	0.30(0.26)	0.29(0.30)	0.34(0.31)	0.23(0.12)	0.23(0.23)	0.26(0.26)	0.27(0.27)	0.13(0.10)
Site-stack	0.23(0.27)	0.10(0.30)	0.33(0.29)	0.15(0.16)	0.20(0.22)	0.04(0.19)	0.30(0.27)	0.19(0.14)
Biased site-stack	0.28(0.26)	0.26(0.28)	0.32(0.27)	0.15(0.16)	0.22(0.24)	0.11(0.24)	0.30(0.26)	0.19(0.14)

Supplementary Table 2 Radiative effect (unit: W m^{-2}) at the top of atmosphere due to different processes when dust is removed in both MH and EH. The values are averaged over the globe except for dust and heat transport, which are divided into the northern hemisphere, tropics and southern hemisphere.

	100% dust removal				Atm.+ocn. heat transp			water vapor	cloud	albedo	Temp
	Global	N. Hemi.	Tropics	S. Hemi	N. Hemi.	Tropics	S. Hemi				
MH	0.68	0.54	1.04	0.08	0.09	-0.16	0.23	0.58	-0.20	0.06	-1.02
EH	0.68	0.53	1.05	0.08	0.14	-0.13	0.11	0.45	-0.31	0.05	-0.80

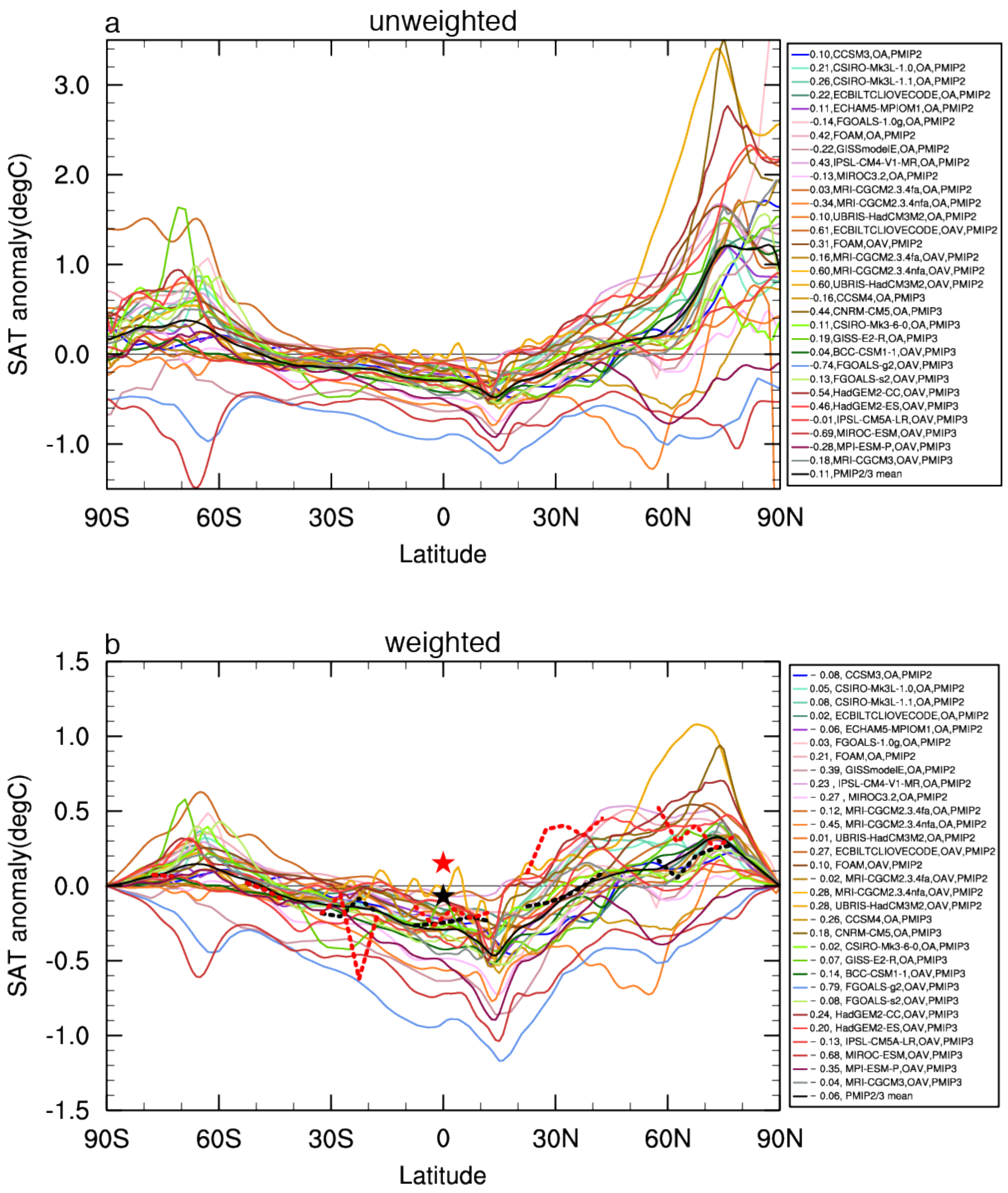

Supplementary Figure 1 Zonal-mean surface temperature changes of mid-Holocene (6ka) relative to that of preindustrial obtained by all models participated in PMIP2[2] and PMIP3. a) unweighted; b) weighted by the area of the corresponding latitudinal band with the band area at equator assumed to be 1. Ensemble mean of all the models is shown by the black curve, which has a global

mean annual temperature (anomaly) of -0.06 °C. When a virtual site stack is created from the model data in the same way as for the proxy data[3], the zonal mean and global mean are shown by the black dashed curve and black pentacle, respectively, where the global mean value is also -0.06 °C. If the seasonal bias of the proxy data is considered in constructing the site stack, the zonal mean and global mean are shown by the red dashed curve and red pentacle, respectively. The most obvious change in the red dashed curve relative to the black dashed curve is in the northern hemisphere, there is no change in the southern hemisphere. The global mean surface temperature anomaly is now 0.16 °C, which is still much smaller than the ~0.5 °C required by the real proxy data. Note that the concentrations of greenhouse gases in Liu et al.[1] are time dependent, and are lower at 9 ka and 6 ka than PI values, while they are very similar between the 6 ka and PI in the PMIP experiments.

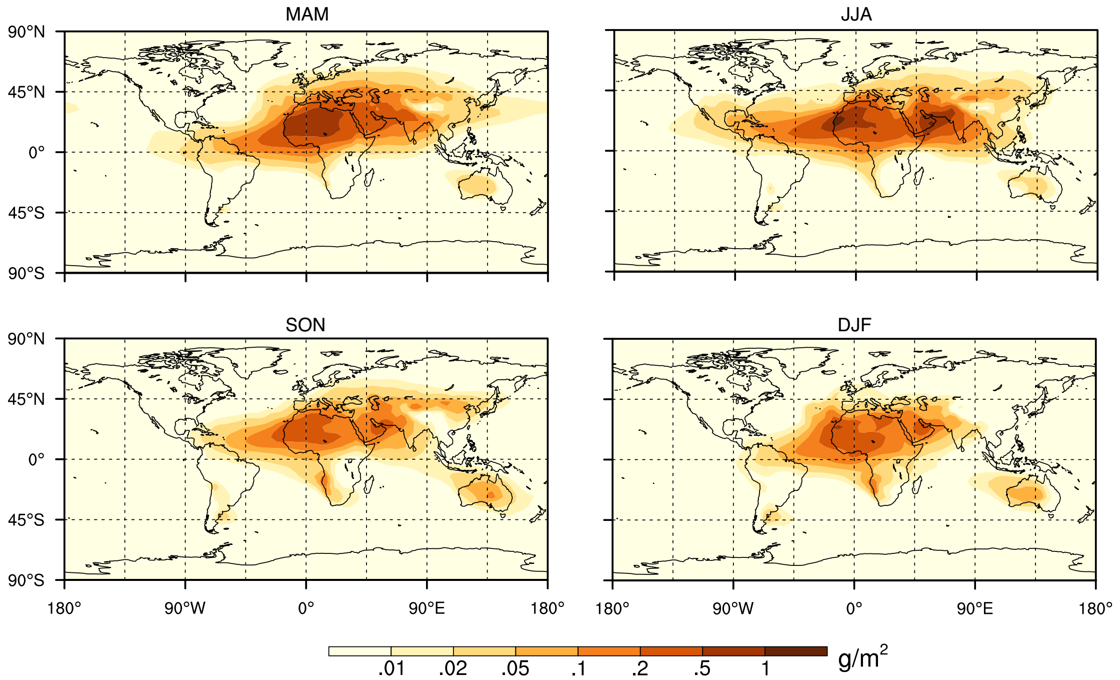

Supplementary Figure 2 Total mass of dust in the air column for the four seasons of the present-day climate. Note the nonlinear color scale. Figure generated using NCL[4].

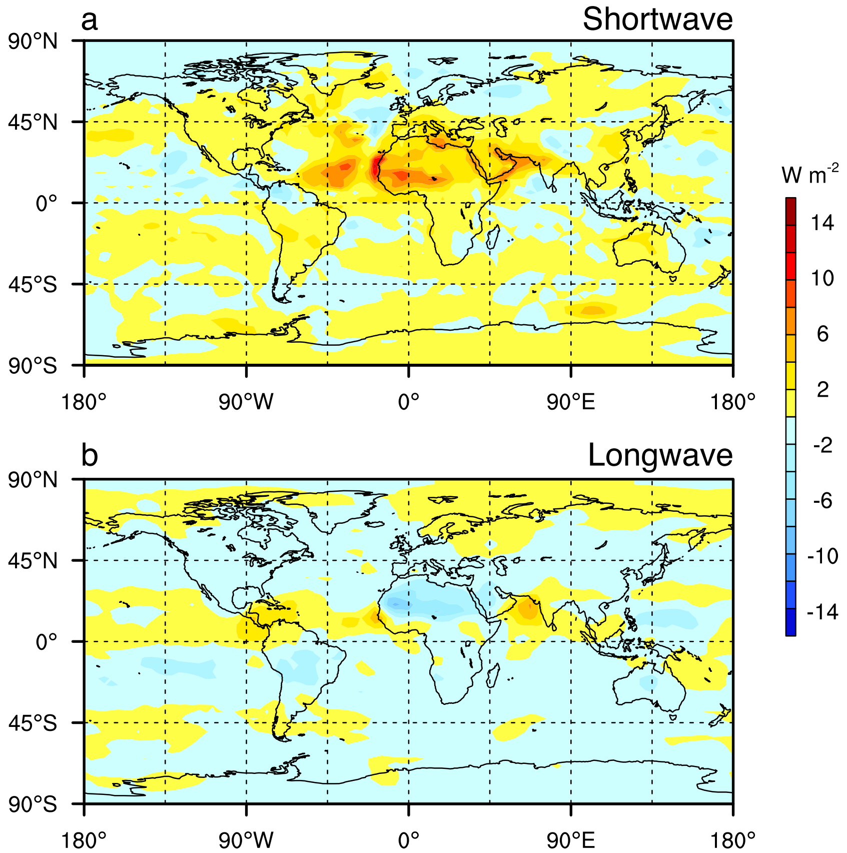

Supplementary Figure 3 Change of net a) shortwave and b) longwave radiative fluxes at top-of-atmosphere for the MH climate when dust is completely removed. Figure generated using NCL[4].

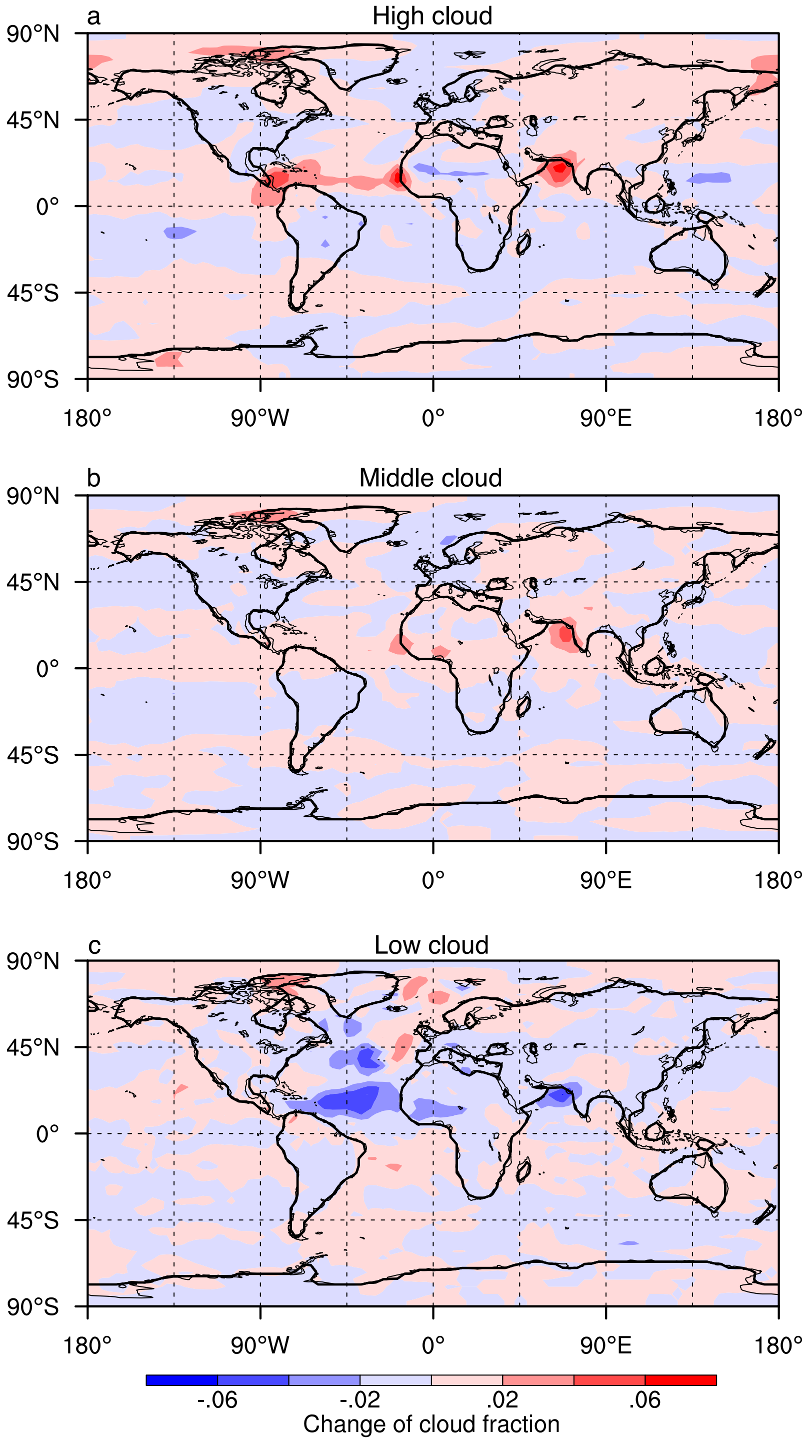

Supplementary Figure 4 Change of a) high (<400 hPa), b) middle (between 400 and 700 hPa) and c) low cloud (>700 hPa) fraction for the MH (6ka) climate when dust is completely removed. Figure generated using NCL[4].

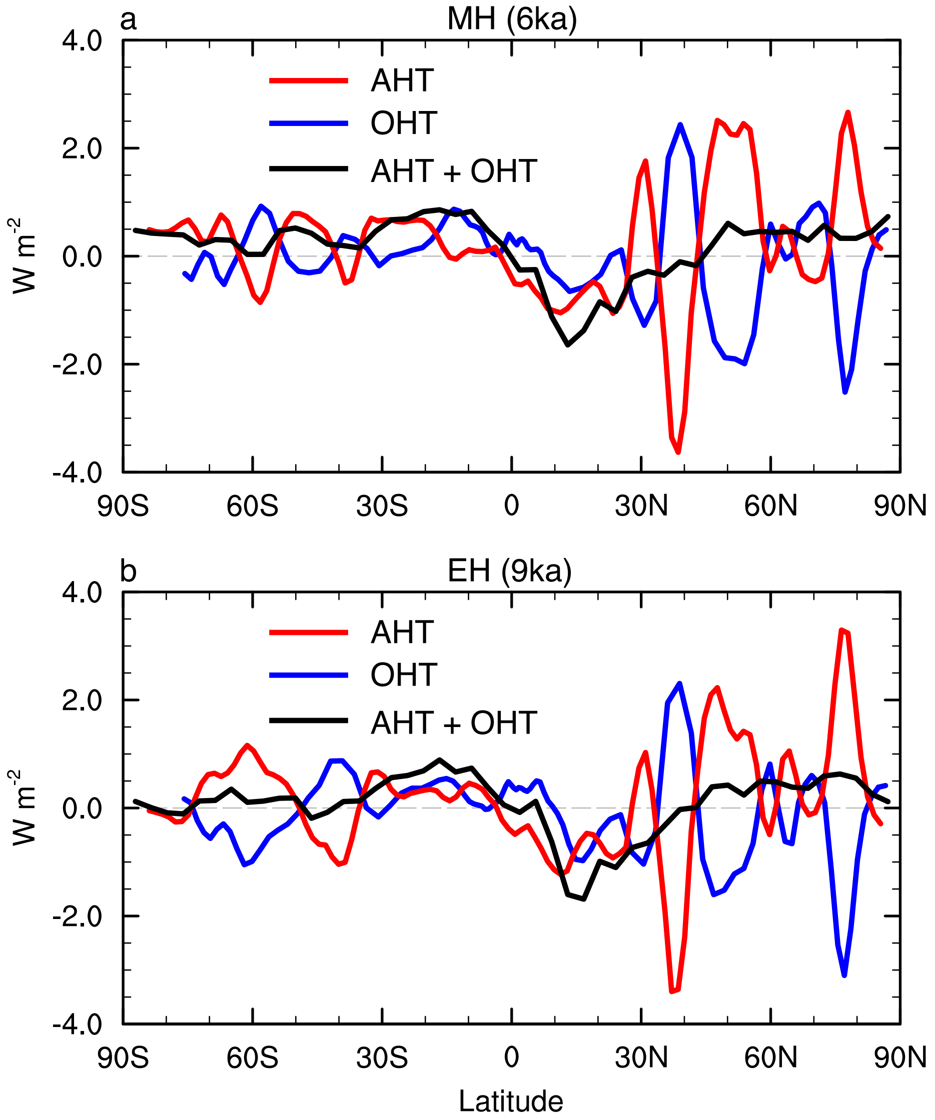

Supplementary Figure 5 Equivalent radiative forcing due to changes of atmospheric heat transport (AHT; red) and oceanic heat transport (OHT; blue) when dust is completely removed. The total heat transport is shown in black. a) MH; b) EH. Figure generated using NCL[4].

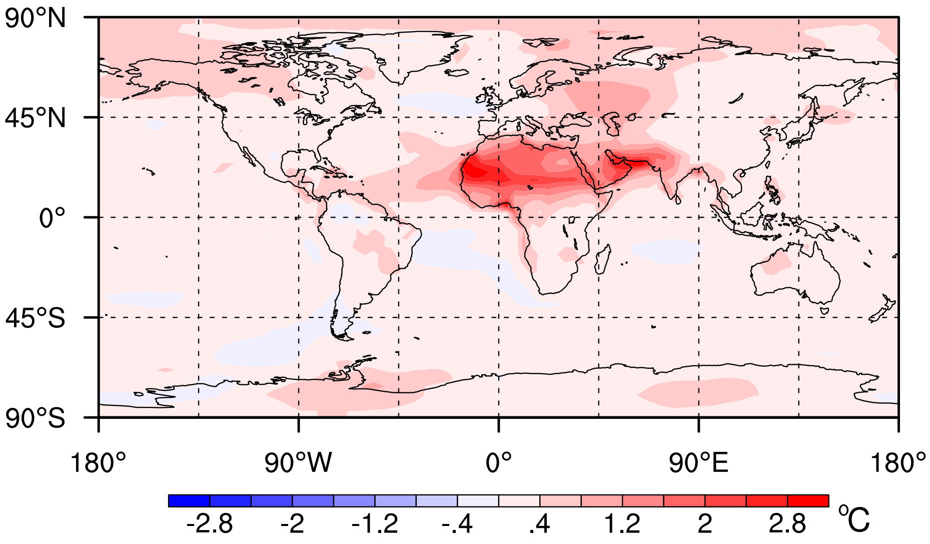

Supplementary Figure 6 Change of surface temperature for MH (6ka) when dust is completely removed, obtained with the atmospheric component of CCSM3, CAM3, coupled to a 50 m slab ocean. The model is run for 180 years, and the result shown here is the average of the last 60 years. Figure generated using NCL[4].

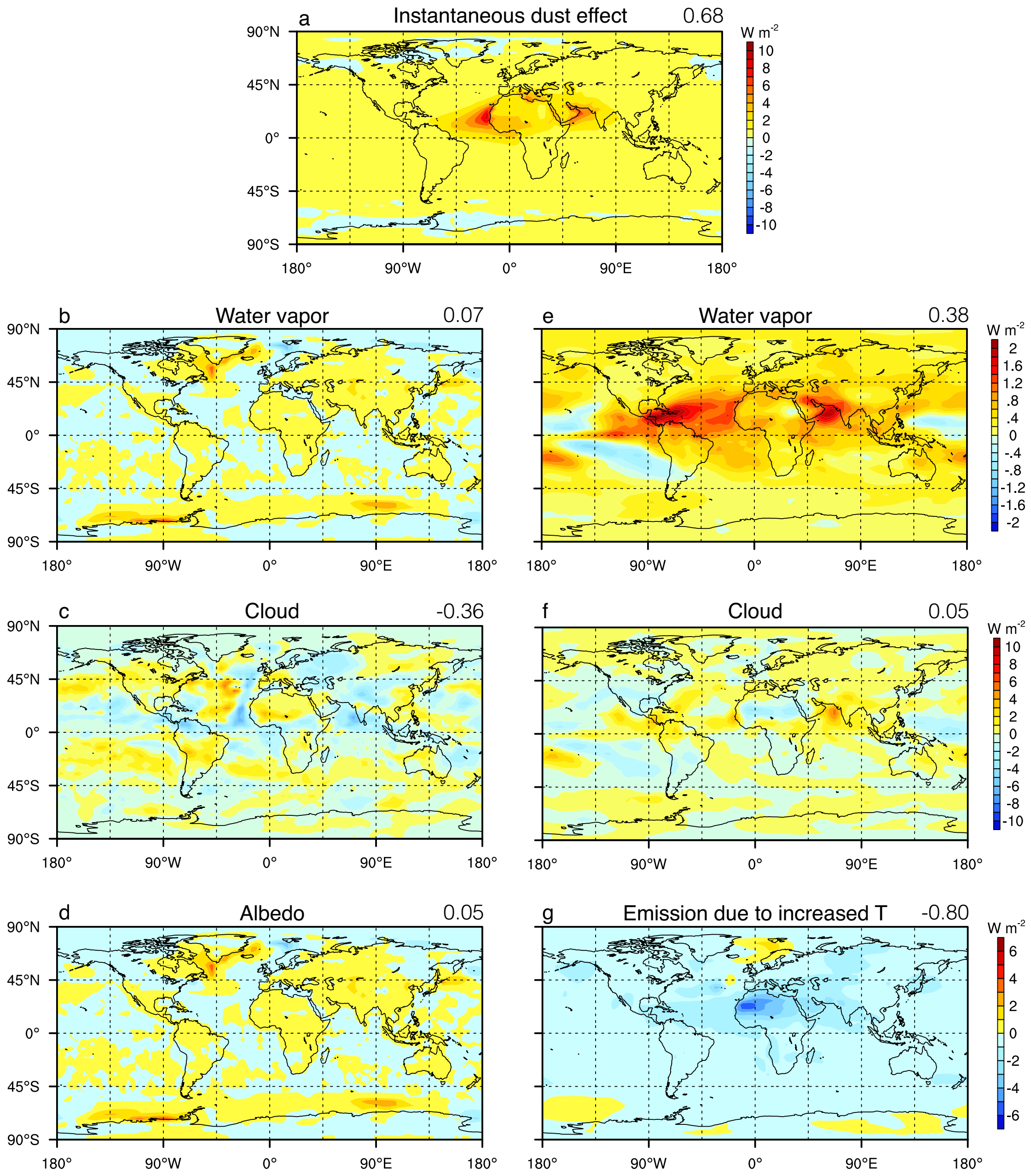

Supplementary Figure 7 Change of shortwave (a - d) and longwave (e - g) top-of-atmosphere radiative fluxes due to different components for the EH climate when dust is completely removed. In g), the increased outward longwave emission is due to temperature increase of both surface and atmosphere. The global mean values are indicated at the top right corner of each panel, with unit of W m^{-2}. Note the different color scale for each row. Figure generated using NCL

[4].

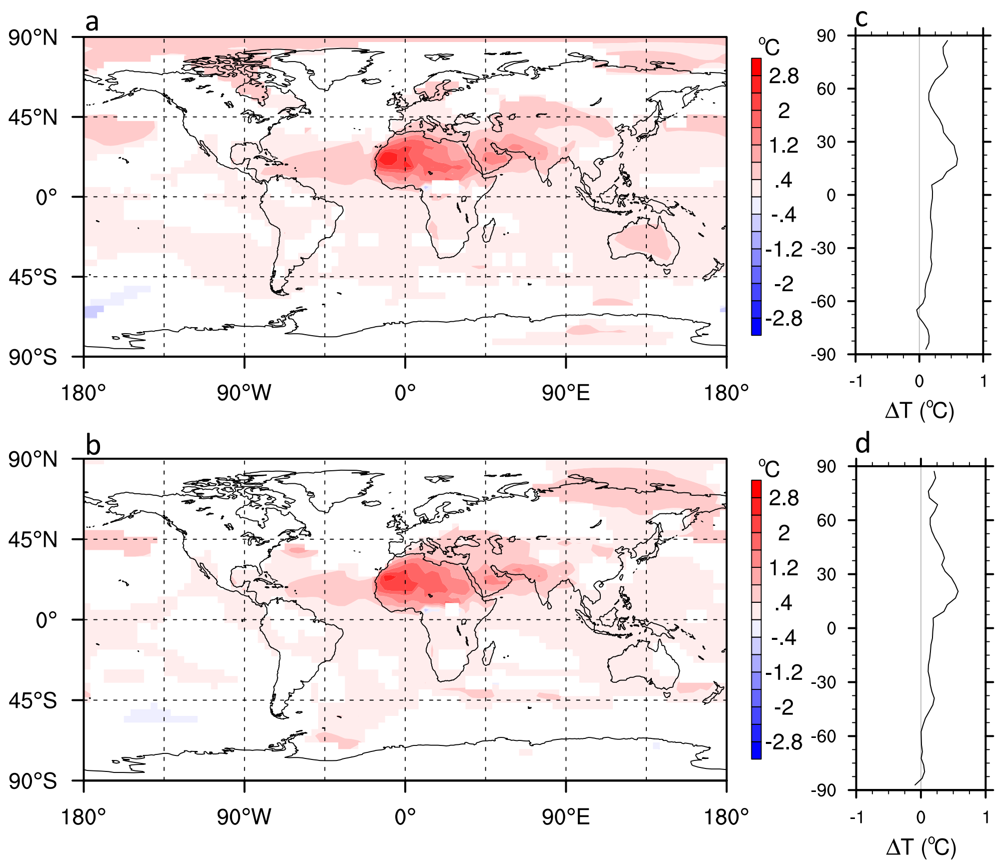

Supplementary Figure 8 The surface temperature change in a) 6 ka and b) 9 ka when North Africa is changed to grassland and global dust is completely removed. Only the changes that are significant to the 5% level (calculated by two-tailed student-t test) are shown. The zonal-mean temperature change in a) and b) are shown in c) and d), respectively. Figure generated using NCL[4].

References

1 Liu, Z. *et al.* The Holocene temperature conundrum. *P Natl Acad Sci USA* **111**, E3501-E3505 (2014).

2 Braconnot, P. *et al.* Results of PMIP2 coupled simulations of the Mid-

Holocene and Last Glacial Maximum - Part 1: experiments and large-scale features. *Clim Past* **3**, 261-277 (2007).
3 Marcott, S. A., Shakun, J. D., Clark, P. U. & Mix, A. C. A Reconstruction of Regional and Global Temperature for the Past 11,300 Years. *Science* **339**, 1198-1201 (2013).
4 The NCAR Command Language (Version 6.3.0) [Software]. *Boulder, Colorado: UCAR/NCAR/CISL/TDD*, http://dx.doi.org/10.5065/D5066WD5063XH5065 (2015).
